# Flurbiprofen Ameliorates Glucose Deprivation-Induced Leptin Resistance

**DOI:** 10.3389/fphar.2016.00354

**Published:** 2016-09-30

**Authors:** Toru Hosoi, Yuka Suyama, Takaaki Kayano, Koichiro Ozawa

**Affiliations:** Department of Pharmacotherapy, Graduate School of Biomedical and Health Sciences, Hiroshima UniversityHiroshima, Japan

**Keywords:** leptin, STAT3, STAT5, AMPK, glucose, neuron

## Abstract

Leptin resistance is one of the mechanisms involved in the pathophysiology of obesity. The present study showed that glucose deprivation inhibited leptin-induced phosphorylation of signal transducer and activator of transcription 3 (STAT3) and signal transducer and activator of transcription 5 (STAT5) in neuronal cells. Flurbiprofen reversed glucose deprivation-mediated attenuation of STAT3, but not STAT5 activation, in leptin-treated cells. Glucose deprivation increased C/EBP-homologous protein and glucose regulated protein 78 induction, indicating the activation of unfolded protein responses (UPR). Flurbiprofen did not affect the glucose deprivation-induced activation of UPR, but did attenuate the glucose deprivation-mediated induction of AMP-activated protein kinase phosphorylation. Flurbiprofen may ameliorate glucose deprivation-induced leptin resistance in neuronal cells.

## Introduction

Leptin is an anti-obesity hormone that attenuates food intake and enhances energy expenditure (Zhang et al., 1994; Campfield et al., 1995). It exerts anti-obesity action through Ob-Rb, the long isoform of the leptin receptor that is expressed mainly in hypothalamic neuronal cells (Fei et al., 1997). Leptin activates both the Janus kinase 2 (JAK2)-signal transducer and activator of transcription 3 (STAT3) and JAK2-STAT5 pathways upon activation of the Ob-Rb leptin receptor (Ghilardi et al., 1996; Vaisse et al., 1996; Hosoi et al., 2002; Gong et al., 2007; Mütze et al., 2007). Because leptin has an anti-obesity effect, the hormone was initially expected to decrease body weight in obese patients. However, the majority of obese patients are leptin-resistant, and do not respond to leptin efficiently (Heymsfield et al., 1999). Therefore, though leptin is now thought to be unsuccessful for the treatment of obesity, understanding the mechanisms of the development of “leptin resistance” has become a hot topic for research (Friedman and Halaas, 1998; Friedman, 2003). Several mechanisms of the pathogenesis have been proposed (Hosoi and Ozawa, 2010); we and other research groups have recently suggested the possible involvement of ER stress (Hosoi et al., 2008; Ozcan et al., 2009). The ER plays a key role in maintaining protein folding. However, when unfolded proteins accumulate in the ER in response to stressful stimuli, cells activate the UPR to cope with the stress (Walter and Ron, 2011). Upon induction of ER stress, cells induce CHOP and GRP78 as one of the UPR (Walter and Ron, 2011). Previously, a candidate drug was identified to ameliorate ER stress-induced leptin resistance: flurbiprofen, a NSAID (Hosoi et al., 2014). However, the action mechanisms of flurbiprofen on leptin have not yet been elucidated in detail.

The hypothalamic neuronal cells senses nutrients, such as glucose, in the blood and regulates energy balance. Glucose up-regulates POMC in the hypothalamic neuron, thereby inhibiting food intake (Zhang et al., 2011), whereas feeding is increased when the glucose level is lowered by 2-deoxy-D-glucose in the rat brain (Miselis and Epstein, 1975). Under low glucose conditions, AMPK is activated. AMPK is a serine-threonine kinase that is activated when the AMP/ATP ratio increases. Glucose (25 mM) enhances leptin signaling by inhibiting AMPK, while AMPK activation under low glucose conditions (5 mM) inhibits leptin signaling (Su et al., 2012). In addition to AMPK, glucose deprivation induced the UPR (Hosoi et al., 2013). Given the important role of glucose on neuronal function in regulating food intake, it is of interest to analyze the pharmacological effect of flurbiprofen on regulating neuronal function in the glucose-deprived state, i.e., leptin-induced STAT3 activation, AMPK activation in neuronal cells.

The crosstalk between nutrients and hormone action plays an important role in regulating neuronal activity, which subsequently affects the feeding response. As the flurbiprofen has anti-obesity effect (Hosoi et al., 2014), it is of interest to analyze the effect of flurbiprofen on the regulation of glucose mediated neuronal activity, which regulates energy homeostasis. In the present study, we investigated the effect of flurbiprofen on leptin action in the glucose-deprived state in neuronal cells.

## Materials and Methods

### Reagents

Flurbiprofen was obtained from Cayman Chemical (MI). Human leptin was obtained from Sigma (L4146; St. Louis, MO, USA) or ENZO Life Sciences (SE-161; Plymouth Meeting, PA, USA).

### Generation of Ob-Rb Leptin Receptor-Transfected Cells

The human Ob-Rb leptin receptor construct was a kind gift from Genetech, Inc. (CA). The construct was transfected into SH-SY5Y cells using the LipofectAMINE PLUS Reagent (Life Technologies, Inc.) according to the manufacturer’s instructions. The stable transfectants were obtained by selection with the antibiotic G418 (Hosoi et al., 2006).

### Cell Culture

Human neuroblastoma SH-SY5Y-Ob-Rb cells were maintained in Dulbecco’s modified Eagle’s medium supplemented with 10% (v/v) heat-inactivated fetal calf serum at 37°C in a humidified incubator under 5% CO_2_ and 95% air. We treated the cells with flurbiprofen at the concentration of 100 μM in the present experiment.

### Western Blotting Analysis

Western blotting was performed as described previously (Hosoi et al., 2015). Briefly, cells were washed with ice-cold PBS and lysed in buffer containing 10 mM HEPES-NaOH (pH 7.5), 150 mM NaCl, 1 mM EGTA, 1 mM Na_3_VO_4_, 10 mM NaF, 10 μg/ml aprotinin, 10 μg/ml leupeptin, 1 mM phenylmethylsulfonyl fluoride (PMSF), and 1% NP-40 for 20 min. The lysates were centrifuged at 15,000 rpm for 20 min at 4°C, and supernatants were collected. Samples were boiled with Laemmli buffer for 3 min, fractionated by sodium dodecylsulfate-polyacrylamide gel electrophoresis (SDS-PAGE), and transferred at 4°C to nitrocellulose membranes. These membranes were then incubated with anti-phospho STAT3 (Tyr705: Cell Signaling; 1:1,000), anti-STAT3 (Santa Cruz; 1:1,000), anti-phospho STAT5 (Tyr694: Cell Signaling; 1:1,000), and anti-GAPDH (Chemicon; 1:1,000) antibodies, followed by an anti-horseradish peroxidase-linked antibody. Peroxidase binding was detected by chemiluminescence using an enhanced chemiluminescence system (Thermo scientific). Multiple independent experiments were performed and the numbers of the experiments performed was indicated in the figure legends (*n* = 3∼7).

### Gene Expression Analysis

Total RNA was isolated using the TriPure Isolation Reagent (Roche Molecular Biochemicals, Indianapolis, IN, USA). cDNA was synthesized from 2 μg of total RNA by reverse transcription using ReverTra Ace (Toyobo, Japan), Oligo (dt)_16_ primer (SP230; Eurofins, Japan) in a 20 μl reaction mixture containing RT buffer (Toyobo, Japan), 1 mM dNTP mix, 10 mM dithiothreitol (DTT), and 40 U of RNase inhibitor (Y9240L; Enzymatics, Beverly, MA, USA). Total RNA and the Oligo (dt)_16_ primer were pre-incubated at 70°C for 10 min prior to the reverse transcription. After incubation for 1.5 h at 46°C, the reaction was terminated by incubating samples for 5 min at 100°C. For PCR amplification, 1.2 μl of cDNA was added to 10.8 μl of a reaction mix containing 0.2 μM of each primer, 0.2 mM of dNTP mix, 0.6 U of Taq polymerase (3300226001; Expand High Fidelity^PLUS^ PCR System, Roche Diagnostics, Switzerland). The following primer sequences were used: GRP78; upstream, 5′-TGC TTG ATG TAT GTC CCC TTA-3′, and downstream, 5′-CCT TGT CTT CAG CTG TCA CT-3′, CHOP; upstream, 5′-GCA CCT CCC AGA GCC CTC ACT CTC C-3′, and downstream, 5′-GTC TAC TCC AAG CCT TCC CCC TGC G-3′, GAPDH; upstream, 5′-AAA CCC ATC ACC ATC TTC CAG-3′ and downstream, 5′-AGG GGC CAT CCA CAG TCT TCT-3′. The PCR products (10 μl) were resolved by electrophoresis using an 8% polyacrylamide gel. The gel was stained with ethidium bromide and photographed under ultraviolet light. The density of each band was measured using Image J 1.37v (Wayne Rasband, NIH) software. Multiple independent experiments were performed and the numbers of the experiments performed was indicated in the figure legends (*n* = 3∼4).

### Statistics

Results are expressed as the mean ± SE of the stated value. Statistical analyses were performed using the Student’s *t*-test.

## Results

### Flurbiprofen Specifically Ameliorated Glucose Deprivation-Induced Impairment of STAT3 but not STAT5 in Leptin-Treated Cells

**FIGURE 1:**
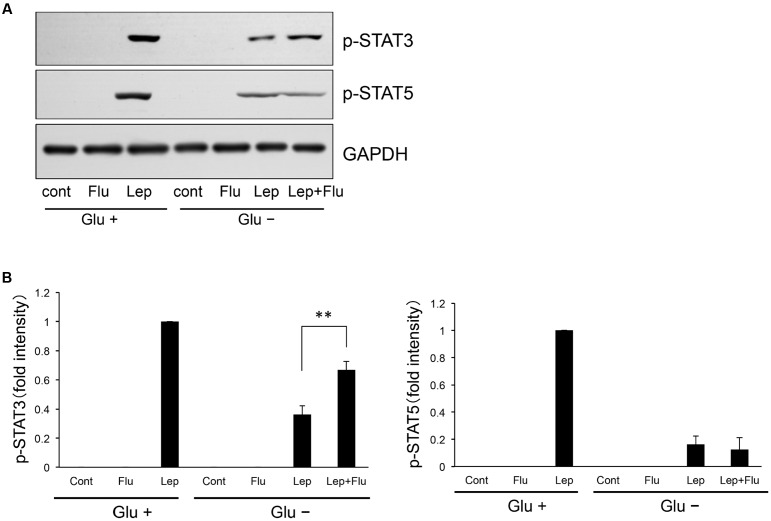
**Flurbiprofen ameliorates inhibition of leptin-induced STAT3 but not STAT5 signaling in the glucose deprivation state.** **(A)** SH-SY5Y Ob-Rb cells were treated with flurbiprofen (Flu:100 μM) in the absence of glucose for 4 h. Leptin (Lep: 0.5 μg/mL, 15 min)-induced STAT3 and STAT5 phosphorylation was then analyzed by Western blotting. **(B)** Densitometric analyses of phospho-STAT3 and phospho-STAT5 were conducted using image analysis software. Each set of data was expressed as fold increase over leptin treatment (Glu+) cells. ^∗∗^*P* < 0.01 leptin (Glu-) versus leptin+flurbiprofen (Glu-). *n* = 7.

We investigated whether flurbiprofen could ameliorate glucose deprivation-induced leptin resistance in neuronal cells. Leptin (0.5 μg/mL, 15 min) was administered to a SH-SY5Y human neuroblastoma cell line stably transfected with Ob-Rb leptin receptor (SH-SY5Y Ob-Rb), and the cells were analyzed for STAT3 and STAT5 activation. As shown in **Figure 1**, leptin activated both STAT family proteins; i.e., STAT3 and STAT5 (**Figure 1**). Furthermore, glucose deprivation for 4 h impaired the phosphorylation of STAT3 and STAT5 (**Figure 1**). No visible cellular toxicity was observed at these conditions. Therefore, we then analyzed whether flurbiprofen could ameliorate glucose deprivation-induced impairment of STAT3 and STAT5 phosphorylation. Interestingly, flurbiprofen (100 μM) ameliorated the glucose deprivation-induced impairment of STAT3, but not STAT5, phosphorylation in leptin (0.5 μg/mL, 15 min)-treated cells (**Figure 1**). Therefore, flurbiprofen could ameliorate leptin resistance by specifically regulating STAT3.

### Flurbiprofen Did Not Affect Glucose Deprivation-Induced Induction of UPR

**FIGURE 2:**
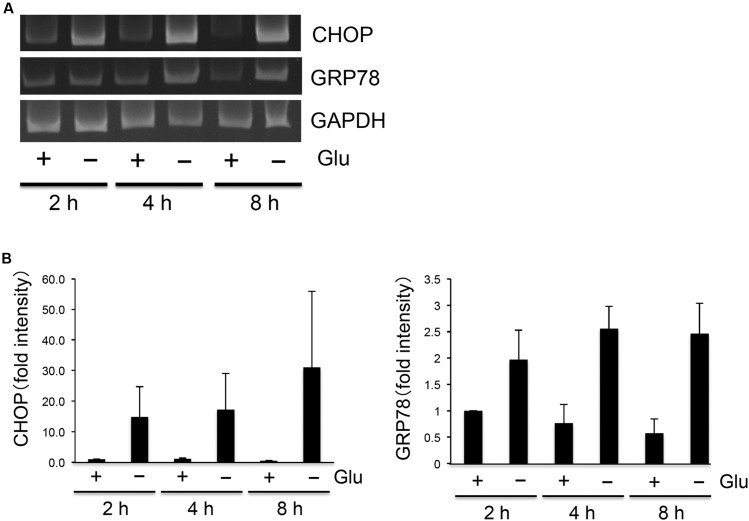
**Glucose deprivation-induced UPR in SH-SY5Y Ob-Rb neuronal cells.** **(A)** SH-SY5Y Ob-Rb cells were cultured in the presence or absence of glucose for 2, 4, and 8 h. ER stress markers, CHOP and GRP78, were analyzed by RT-PCR using specific primers. **(B)** Densitometric analyses of CHOP and GRP78 were conducted using image analysis software. Each set of data was expressed as fold increase over control cells. *n* = 4.

**FIGURE 3:**
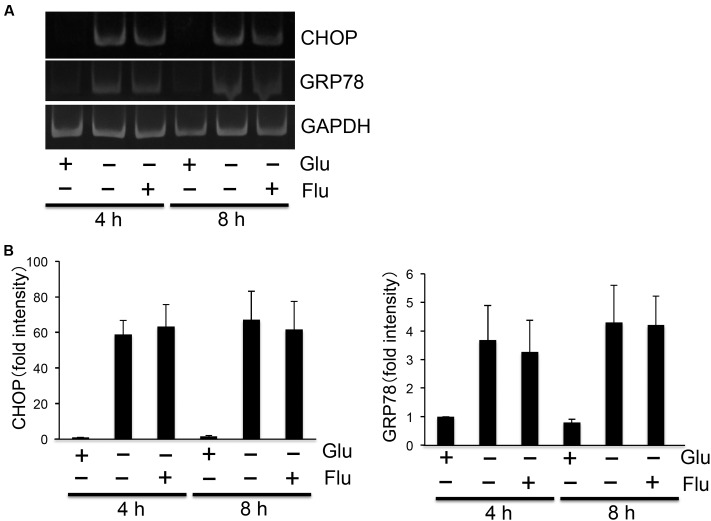
**Flurbiprofen did not affect glucose deprivation-induced induction of UPR.** **(A)** SH-SY5Y Ob-Rb cells were treated with flurbiprofen (Flu:100 μM) in the absence of glucose for 4 and 8 h. ER stress markers, CHOP and GRP78, were analyzed by RT-PCR. **(B)** Densitometric analyses of CHOP and GRP78 were conducted using image analysis software. Each set of data was expressed as fold increase over control cells. *n* = 3.

As flurbiprofen was reported to reduce ER stress (Hosoi et al., 2014), we next analyzed the pharmacological effect of flurbiprofen on regulating glucose deprivation-induced activation of the UPR. Glucose deprivation time-dependently (2, 4, 8 h) induced CHOP and GRP78 in SH-SY5Y Ob-Rb human neuroblastoma cells (**Figure 2**). Therefore, we next analyzed whether flurbiprofen was able to affect the glucose deprivation-induced activation of the UPR. Cellular medium was replaced to glucose-free medium; flurbiprofen was then added and incubated for 4 or 8 h, and the expression levels of CHOP and GRP78 were analyzed. As shown in **Figure 3**, no changes in CHOP or GRP78 levels were observed in flurbiprofen (100 μM)-treated cells. These results suggest that flurbiprofen may not affect the induction of the UPR caused by glucose deprivation in neuronal cells.

### Flurbiprofen Inhibited Glucose Deprivation-Induced Induction of AMPK Phosphorylation

**FIGURE 4:**
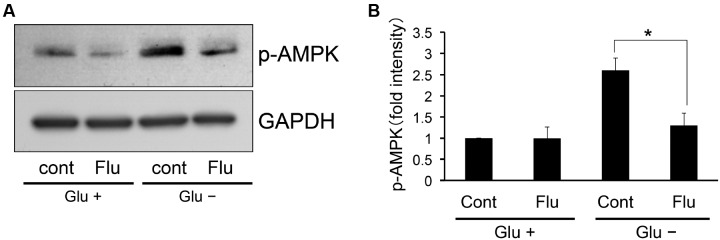
**Flurbiprofen reduces AMPK phosphorylation in the glucose deprivation state.** **(A)** SH-SY5Y Ob-Rb cells were treated with flurbiprofen (Flu:100 μM) in the absence of glucose for 4 h. Phosphorylation status of AMPK was analyzed by Western blotting. **(B)** Densitometric analysis of phospho-AMPK was conducted using image analysis software. ^∗^*P* < 0.05 control (Glu-) versus flurbiprofen (Glu-). Each set of data was expressed as fold increase over control cells. *n* = 3.

AMP-activated protein kinase is activated under low glucose conditions (Kahn et al., 2005). Furthermore, this activation is involved in the pathogenesis of leptin resistance (Su et al., 2012). Therefore, we investigated whether flurbiprofen affects AMPK activation under this condition. Flurbiprofen was administered with or without glucose for 4 h and the phosphorylation status of AMPK was analyzed. We observed that the phosphorylation level of AMPK increased in the glucose-deprived state in SH-SY5Y Ob-Rb neuronal cells; however, treatment with flurbiprofen (100 μM) ameliorated AMPK phosphorylation (**Figure 4**). Therefore, flurbiprofen may be involved in the inhibition of AMPK activation in the glucose-deprived state.

## Discussion

Obesity is one of the major risk factor of metabolic diseases and a lot of effort was made to prevent the disease (Hosoi and Ozawa, 2010; Carter et al., 2016; Scarpace et al., 2016; Strehler et al., 2016). In the present study, we found that flurbiprofen has a unique pharmacological effect on the regulation of neuronal function in the glucose-deprived state. Flurbiprofen can ameliorate glucose deprivation-induced leptin resistance by specifically regulating STAT3, but not STAT5, phosphorylation. Furthermore, while flurbiprofen did not modulate the glucose deprivation-induced activation of the UPR in neuronal cells, it did modulate AMPK activation in the glucose-deprived state. The results of the present study suggest an important pharmacological action of flurbiprofen in regulating neuronal function during glucose deprivation.

Glucose-sensing neurons in the CNS play an important role in maintaining energy homeostasis (Campfield and Smith, 2003). Defects in the response of glucose-sensing neurons are linked with diseases such as obesity and diabetes (Routh, 2010). Therefore, identifying candidate drugs that can regulate the responses of glucose-sensing neurons is important. Interestingly, leptin action was reported to be regulated by glucose, with glucose dose-dependently increasing leptin signaling and glucose deprivation attenuating leptin-induced signaling (Su et al., 2012). In the present study, we found that flurbiprofen increases leptin action in neurons during glucose deprivation by modulating AMPK activity. These results suggest that flurbiprofen has the novel pharmacological action of ameliorating leptin resistance possibly by regulating AMPK-mediated glucose metabolism. It is an important future subject to analyze the effect of flurbiprofen on leptin’s action at more physiological conditions.

In a previous study, we found that flurbiprofen has chemical chaperon activity and could attenuate ER stress by decreasing unfolded protein accumulation in the ER (Hosoi et al., 2014). However, in the present study, we did not observe an inhibition of glucose deprivation-induced activation of the UPR in flurbiprofen-treated cells. We speculate these differences would be due to the different mechanisms of UPR induction between ER stress-inducing reagent- and glucose deprivation-induced activation of UPR. Recently, the ER stress sensor protein has been suggested to play an important role in regulating physiological responses (Rutkowski and Hegde, 2010). For example, it is suggested that ER stress sensor protein would be activated by physiological stimulant independently through the unfolded protein accumulation in the ER (Karali et al., 2014). It is speculated that glucose deprivation-induced activation of UPR would also be mediated independently through the unfolded protein accumulation in the ER. Thus, it is possible that the mechanisms of ER stress-induced activation of UPR are different from those of glucose deprivation-induced activation of the UPR, and therefore, flurbiprofen’s action on UPR regulation yielded different results. It would be interesting to further analyze the mechanisms of flurbiprofen’s pharmacological action in neuronal cells.

In the present study, we elucidated a unique pharmacological property of flurbiprofen: the attenuation of leptin resistance under glucose-deprivation. As the glucose in the hypothalamic neurons may play a role in regulating energy homeostasis, current finding may represent one of a key pharmacological tool for attenuating obesity.

## Author Contributions

TH and KO designed research; YS and TK performed research; TH, YS, and TK analyzed data; TH wrote the paper.

## Conflict of Interest Statement

The authors declare that the research was conducted in the absence of any commercial or financial relationships that could be construed as a potential conflict of interest.
